# Importance of Oral Hygiene Habits in Mentally Disabled Children

**DOI:** 10.5005/jp-journals-10005-1052

**Published:** 2010-04-15

**Authors:** Usha Mohan Das, Beena JP, Divya Reddy

**Affiliations:** 1 Principal, Professor and Head, Department of Pedodontics and Preventive Dentistry,VS Dental College and Hospital, Bengaluru Karnataka, India; 2 Senior Lecturer, Department of Pedodontics and Preventive Dentistry,VS Dental College and Hospital, Bengaluru, Karnataka India; 3 Postgraduate Student, Department of Pedodontics and Preventive Dentistry, VS Dental College and Hospital, Bengaluru Karnataka, India

**Keywords:** Mentally disabled, oral hygiene, gingival hyperplasia.

## Abstract

**Background:**

The main factor related to gingival/periodontal problems in disabled individuals is the inadequacy of the plaque removal from the teeth. Motor coordination problems and muscular limitation in neuromuscularly disabled individuals along with the difficulty in understanding the importance of oral hygiene in mentally disabled individuals have resulted in the progression of inflammatory diseases.

**Case report:**

This report describes a case of cerebral palsy child who developed gingival hyperplasia due to poor oral hygiene practices which remarkably improved by proper motivation and adaptation of oral hygiene measures.

**Conclusion:**

It is important that the caretakers especially mother is informed about the importance of maintaining proper oral hygiene and the harmful effects of not doing so. It forms our duty to guide them towards maintaining good oral hygiene and thereby help in improving overall health of these children.

## INTRODUCTION

Handicap is the loss or limitation of opportunities to take part in the normal life of the community on an equal level with others due to physical and social barriers.^1^ Cerebral palsy is one of these neuromuscularly handicaps, which has specific motor skill problems, delay in developmental milestones, as well as physical findings that might include abnormal muscle tonus, reflexes, and persistent infantile reflexes.^2,3^ In disabled individuals the process of developing gingival/periodontal diseases does not differ from nondisabled individuals. There are no differences in prevention of the diseases and the treatment modalities between these groups. The main factor related to gingival/periodontal problems in disabled individuals is the inadequacy of the plaque removal from the teeth. Motor coordination problems and muscular limitation in neuromuscularly disabled individuals along with the difficulty in understanding the importance of oral hygiene in mentally disabled individuals have resulted in the progression of inflammatory diseases.^4-6^

The following report describes a case of cerebral palsy child who developed gingival hyperplasia due to poor oral hygiene practices which remarkably improved by proper motivation and adaptation of oral hygiene measures.

## CASE REPORT

**Fig. 1:**
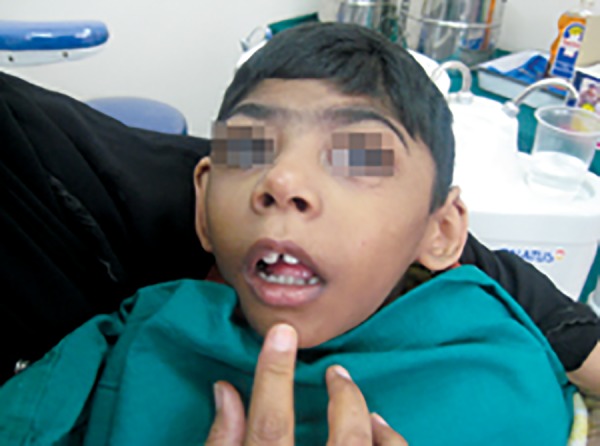
Patient showing cerebral palsy with microcephaly and mental retardation

A male patient aged 6 years reported to Department of Pedodontics and Preventive dentistry, VS Dental College and Hospital, Bengaluru. Patient’s mother complained of severe bleeding from gums from past 1 week and inability of the child to eat. The child is known case of cerebral palsy with microcephaly and mental retardation with developmental age of only a few months (Fig. 1). Patient showed minimal response to verbal commands.

**Fig. 2:**
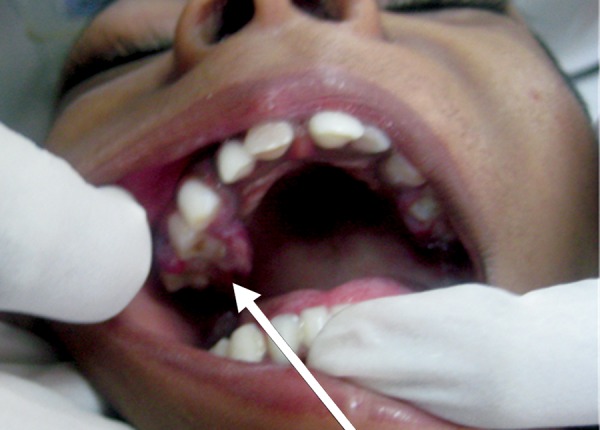
Intraoral photograph showing gingival overgrowth labial and palatal to 54

On intraoral examination, erythematous gingival overgrowth was noted on labial and palatal aspects of 54 (Fig. 2) and labial aspect of 64. On palpation, profuse bleeding was noted.

A detailed case history was taken and it was noted that the patients teeth were cleaned once daily by mother by wiping with a wet cloth. No brushing or use of any dentifrice was done from the beginning.

Depending on clinical examination and based on the oral hygiene status of the patient, a provisional diagnosis of inflammatory gingival hyperplasia due to poor oral hygiene was obtained.

**Fig. 3:**
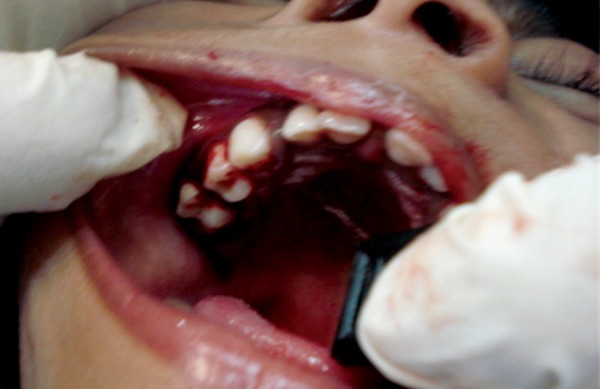
Immediately after surgical excision

**Fig. 4:**
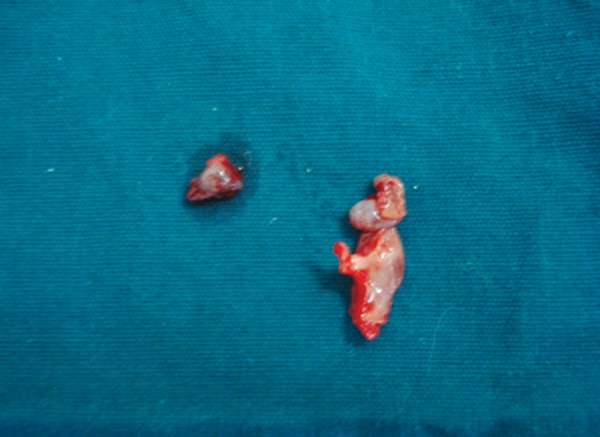
Excision biopsy specimen

Pediatric opinion on general state of patient was obtained and after performing all the necessary blood investigations, excision of hyperplastic gingiva in relation to 54 was planned and done under local anesthesia (Fig. 3). The excisional biopsy specimen was sent for histopathological examination (Fig. 4).

Meanwhile, mother was educated regarding the importance of oral hygiene, method of brushing and other oral hygiene practices. Chlorhexidine mouth rinse was also advised and the surgical excision of the tissue in relation to 64 was deferred till the final diagnosis was obtained.

The histopathological examination confirmed the diagnosis of inflammatory gingival hyperplasia secondary to poor oral hygiene status.

**Fig. 5:**
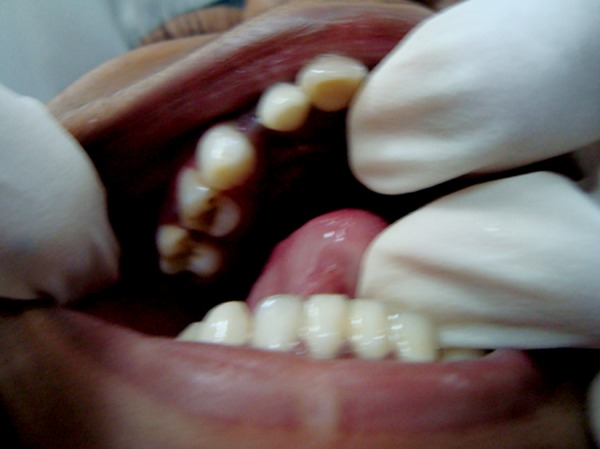
One week after surgery

**Fig. 6:**
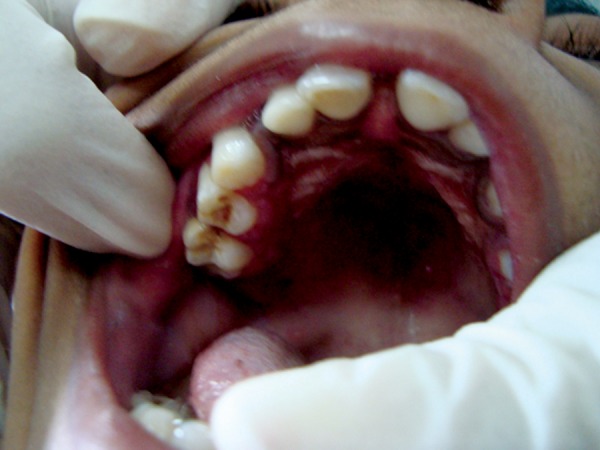
Spontaneous regression on other side

Patient was recalled after 1 week for excision on the other side, i.e in relation to 64 but it was observed that there was spontaneous healing of the gingival tissues probably due to the oral hygiene measures adopted (Figs 5 and 6). Therefore parents were advised to continue with oral hygiene measures and patient is being reviewed regularly.

## DISCUSSION

Oral hygiene practices are voluntary physical activities that have at least two requirements: Motivation and manual dexterity.^7^ Thus, poor oral hygiene is perhaps more prevalent among mentally retarded persons compared to other indi-viduals.^8^ Many studies have found an inverse relationship between the levels of mental retardation and oral hygiene status; the lower the IQ score, the higher the oral hygiene index score.^9,10^ The provision and/or supervision of oral hygiene by parents have been reported to be lower for disabled children than for nondisabled children.^11^ This may lead to various complications like gingival and periodontal diseases, dental caries thereby affecting the nutritional status of the child.

The mechanical control of dental plaque in disabled individuals generally causes some difficulties, is found to be time-consuming, and sometimes ineffective^12,13^ and also the lack of parental education and motivation leads to the development of gingival and periodontal diseases which thereby affects the nutrition status of these children. In this case report, the parents were from low socioeconomic background and were unaware of the importance of oral hygiene practices. No oral hygiene measures were being used or have been used which can be accounted for the gingival hyperplasia.

Various studies have shown that the effectiveness of manual and electric toothbrushes is limited by the manual dexterity and skill of the user.^14^ As a result, chemical plaque control has been recommended as an alternative and adjunc-tive to mechanical plaque control in these special patient groups.^15^ The effectiveness of chlorhexidine (CHX) have widely been investigated in various patient populations, including the disabled, and the results have led CHX to be defined as the “gold standard.”^16-20^ The combination of mechanical and chemical plaque control seemed to be even more beneficial than only using a mouthwash.^21,22^

In the present case, spontaneous healing of the gingival hyperplasia was noted in just 1 week after the adoption of chemical (CHX) and mechanical plaque control measures.

Particularly the education and motivation of parents, especially mother can be considered to be the main factor for the adoption of oral hygiene measures and improved oral hygiene status of the patient.

## CONCLUSION

Oral health problems in mentally disabled children may be because of the parental belief of a reduced importance of oral health in comparison to the over all scheme of health management or maybe more time is devoted to assist these children in other daily activities which are seen to be more important compared to their nondisabled counterparts. It is important that the caretakers especially mother is informed about the importance of maintaining proper oral hygiene and the harmful effects of not doing so. It forms our duty to guide them towards maintaining good oral hygiene and thereby help in improving overall health of these children.
